# Genome mining of cryptic bisabolenes that were biosynthesized by intramembrane terpene synthases from *Antrodia cinnamomea*

**DOI:** 10.1098/rstb.2022.0033

**Published:** 2023-02-27

**Authors:** Ranuka T. Hewage, Cheng-Chung Tseng, Suh-Yuen Liang, Chen-Yu Lai, Hsiao-Ching Lin

**Affiliations:** ^1^ Institute of Biological Chemistry, Academia Sinica, Taipei 115, Taiwan; ^2^ Chemical Biology and Molecular Biophysics Program, Taiwan International Graduate Program, Academia Sinica, Taipei 11529, Taiwan; ^3^ Department of Chemistry, National Taiwan University, Taipei 106, Taiwan; ^4^ Department of Indigenous Medical Resources, Gampaha Wickramarachchi University of Indigenous Medicine, Yakkala 11870, Sri Lanka; ^5^ School of Pharmacy, College of Medicine, National Taiwan University, Taipei 100, Taiwan

**Keywords:** biosynthesis, terpenoids, terpene synthases, bisabolenes, *Antrodia*

## Abstract

Terpenoids represent the largest structural family of natural products (NPs) and have various applications in the pharmaceutical, food and fragrance industries. Their diverse scaffolds are generated via a multi-step cyclization cascade of linear isoprene substrates catalysed by terpene synthases (TPSs). Bisabolene NPs, which are sesquiterpenes (C15), have wide applications in medicines and biofuels and serve as bioactive substances in ecology. Despite the discovery of some canonical class I TPSs that synthesize bisabolenes from plants, bacteria and insects, it remained unknown whether any bisabolene synthases from fungi could produce bisabolenes as a main product. *Antrodia cinnamomea*, a Basidiomycota fungus, is a medicinal mushroom indigenous to Taiwan and a known prolific producer of bioactive terpenoids, but little is known regarding the enzymes involved in the biosynthetic pathways. Here, we applied a genome mining approach against *A. cinnamomea* and discovered two non-canonical UbiA-type TPSs that both synthesize (+)-(*S*,*Z*)-α-bisabolene (**1**). It was determined that two tailoring enzymes, a P450 monooxygenase and a methyltransferase, install a C14-methyl ester on the bisabolene scaffold. In addition, four new bisabolene derivatives, **2** and **4**–**6**, were characterized from heterologous reconstitution in *Saccharomyces cerevisiae*. Our study uncovered enzymatic tools to generate structurally diverse bisabolene NPs.

This article is part of the theme issue ‘Reactivity and mechanism in chemical and synthetic biology’.

## 1. Introduction

Terpenoids are the most structurally diverse family of natural products (NPs) and are widely distributed in all kingdoms of life. They are close to human life and have various applications as flavours (e.g. menthol), food colourants (e.g. carotenoids) and medicines (e.g. the antimalarial agent artemisinin and the antimitotic agent Taxol) [1]. Terpenoids are constructed from five-carbon isoprene units, dimethylallyl diphosphate (DMAPP) and isopentenyl diphosphate, through head-to-tail coupling reactions and to give linear C_5n_ isoprene diphosphates as activated precursors.

The diverse scaffolds of terpenoids, with multiple rings and stereogenic centres, are generated via a multi-step cyclization cascade of linear C_5n_ isoprene substrates by terpene synthases (TPSs) [2]. Over decades, two classes of canonical TPSs have been categorized according to their protein sequences, enzymatic structures and functional mechanisms on how the initial carbocation is generated [3]. Class I TPSs catalyse ionization-initiated cyclization to generate a carbocation by leaving a pyrophosphate group through the conserved DDxxD/E motif. Class II TPSs initiate cyclization by protonation of a double-bond or epoxide moiety in an isoprene substrate via the conserved DxDD (DxDTT) motif.

In recent years, there have been a growing number of non-canonical TPSs that catalyse TPS-like cyclization, in which the enzymes differ in sequence or structure from the canonical TPSs; however, like the canonical TPSs, they catalyse cyclization through the generation of carbocation by taking isoprene-derived molecules as precursors [4]. Exploring and characterizing new non-canonical TPSs will provide more insights for enzymatic tools to generate chemical and structural diversity and to understand how these enzymes evolve for terpenoid biosynthesis.

**Figure 1.**
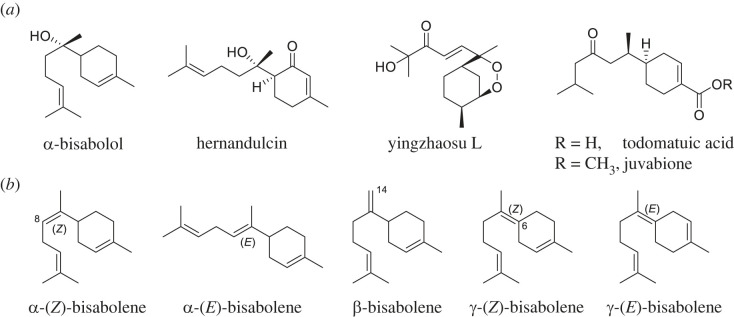
(*a*) NPs derived from the bisabolene scaffold. (*b*) Structure of bisabolene isomers.

Bisabolenes and their derivatives, which belong to sesquiterpenes (C_15_), have wide applications in biofuels and pharmaceuticals and play biological roles in ecology. For example, *α*-bisabolene is a potential biosynthetic substitute for D2 diesel with beneficial fuel properties [5], while *α*-bisabolol is the main constituent of essential oil from chamomile (*Matricaria recutita*), a herbal medicine (figure 1*a*) [6]. Hernandulcin is an intensely sweet molecule that originates from the chief Mexican and South American plant *Lippia dulcis* [7]. In addition, yingzhaosus from the roots of *Artabotrys hexapetalus* exhibit cytotoxicity and antiviral activity against Coxsackie virus B3 and influenza virus A H3N2 [8].

Bisabolene NPs are usually derived from the three isomers *α*-, *β*- and *γ*-bisabolene (figure 1*b*). The bisabolane scaffold is generated from C_15_ farnesyl diphosphate (FPP) catalysed by class I TPSs, which involve an ionization cyclization reaction [2]. FPP cyclization proceeds through C1–C6 bond formation of the initially formed allylic carbocation and gives a bisabolyl cation intermediate (where the first chiral centre at C6 is generated). Deprotonation of either H8, H14 or H6 leads to the formation of *α*-(7*E* or 7*Z*)-, *β*- and *γ*-(7*E* or 7*Z*)-bisabolene, respectively (figure 1*b*).

Plants are common producers of bisabolene NPs, and different plant bisabolene synthases have been characterized, such as (*E*)-*α*-bisabolene synthase [9,10], (*S*)-*β*-bisabolene synthase [11–13] and (*Z*)-*γ*-bisabolene [14]. Some TPSs from insects and bacteria also produce bisabolene scaffolds as main products, such as (+)-(*S*,*Z*)-*α*-bisabolene synthase [15] and sesquipiperitol synthase from harlequin bugs [16] and (*Z*)-*γ*-bisabolene synthase from bacteria [17].

Bisabolenes have been observed to be produced by many Ascomycota and Basidiomycota fungi [18–20]. However, no bisabolene synthases but only other mutated fungal TPSs have been determined to produce bisabolene in small amounts. The trichodiene synthase mutant (D100E) from *Fusarium sporotrichioides* produced (–)-(*Z*)-*α*-bisabolene (11%) and *β*-bisabolene (11%) together with other sesquiterpenes [21], while the *α*-cuprenene synthase (Cop6) mutant (N224D) from *Coprinus cinereus* produced (6*S*)-*β*-bisabolene (18%) as a minor product [22]. It remains unknown whether there is any bisabolene synthase from fungi that produces bisabolenes as the main or even sole product.

Basidiomycota fungi encode more abundant sesquiterpene synthases and UbiA-type prenyltransferases and cyclases than Ascomycota [23–25]. In recent years, some non-canonical TPSs were discovered in Basidiomycota fungi, such as the UbiA-type (–)-cyatha-3,12-diene synthase (EriG) from the mushroom *Hericium erinaceurm* [26]. These results indicate the potential capacity of Basidiomycota to equip unique non-canonical TPSs to create structural diversity.

*Antrodia cinnamomea* (also named *Taiwanofungus camphoratus*) is a medicinal fungus indigenous to Taiwan that is used as a traditional medicine for the treatment of alcohol detoxification, cancer, liver diseases, fatigue and viral infection [27]. The annual commercial market of its fruiting bodies is estimated to be over US$100 million in Taiwan [28]. Terpenoids are the major metabolites of *A. cinnamomea,* such as the sesquiterpene antrocin, the triterpenoid antcins and the meroterpenoid antroquinolols [29]. As the genome of *A. cinnamomea* was published, several class I TPSs and a prenyltransferase have been characterized through heterologous expression in *Escherichia coli* [28,30]. To date, the other enzymatic machineries used by *A. cinnamomea* to synthesize terpenoids remain unclear. In this study, we used a genome mining approach by analysing genes encoding UbiA-type prenyltransferase and discovered two UbiA-type TPSs that both synthesize (+)-(*S*,*Z)*-*α*-bisabolene (**1**). By *in vivo* heterologous expression in *Saccharomyces cerevisiae* and *in vitro* biochemical assays, two modifying enzymes were characterized, including one P450 monooxygenase and one methyltransferase.

## 2. Material and methods

### (a) Strains and culture conditions

The *A. cinnamomea* s27 strain [28] was maintained on PDA (4 g l^−1^ potato inclusion, 29 g l^−1^ dextrose and 15 g l^−1^ agar) and stored as 30% glycerol stocks at –80°C. Total RNA for reverse transcription-polymerase chain reaction (RT–PCR) was extracted from *A. cinnamomea* grown on potato dextrose broth liquid medium after 12 days of cultivation. *Saccharomyces cerevisiae* BJ5464-NpgA for the heterologous experiment was grown in yeast extract peptone dextrose (YPD) medium (10 g l^−1^ yeast extract, 20 g l^−1^ peptone and 10 g l^−1^ glucose) at 28°C and shaken at 250 rpm.

### (b) Chemicals and chemical analysis

For the analysis of chemical metabolic profiles, *S. cerevisiae* transformants were cultured in YPD at 28°C for 5 days. The cell and medium parts were obtained by centrifugation and extracted with acetone and ethyl acetate, respectively. The extracts were dried and dissolved in methanol and analysed by liquid chromatography-diode array detection-mass spectrometry (LC-DAD-MS). All LC-MS analyses were performed on a Shimadzu 2020 LC-MS (Kinetex® 2.6 μm Polar C18 100 Å, 100 × 2.1 mm column) using positive and negative mode electrospray ionization (ESI) with a linear gradient of 5–95% acetonitrile to water (with 0.5% formic acid) over 10 min followed by 95% acetonitrile to water for 4 min with a flow rate of 0.5 ml min^−1^. All gas chromatography-mass spectrometry (GC-MS) analyses were performed on an Agilent 7890B GC with a 5977B mass selective detector and a PAL RTC autosampler with a DB-FFAP column (30 m, 0.32 mm i.d., 0.25 μm film, Agilent Technologies). A flow rate of 2.2 ml min^−1^ helium was used as the carrier gas, and the electronic impact was 70 eV. The oven temperature was programmed at 80°C for 1 min, from 80°C to 230°C for 15 min and 230°C for 3 min. Sample injections were performed with solid-phase microextraction (SPME). The sample was extracted for 5 min at 80°C and desorbed for 2 min at 240°C. ^1^H, ^13^C and two-dimensional nuclear magnetic resonance (NMR) spectra were obtained on a Bruker AV500 spectrometer with a 5 mm dual cryoprobe at the High Field NMR Center at Academia Sinica. High-resolution-electron ionization mass spectra were collected on a JMS-700 double-focusing mass spectrometer (JEOL, Tokyo, Japan). Optical rotation values were measured with a Jasco P-2000 polarimeter. High-resolution-electrospray ionization (ESI) mass spectra were collected on a JMS-T100LP AccuTOF LC-plus 4G mass spectrometer (JEOL, Tokyo, Japan).

### (c) Heterologous reconstitution of the *tps1* gene cluster in *Saccharomyces cerevisiae*

Different combinations of plasmids were co-transformed into the *S. cerevisiae* RC01 strain [31] (= BJ5464-NpgA harbouring *AtCPR*) by using a frozen-EZ yeast transformation II kit™ (Zymo Research). The five combinations are as follows: (i) pXW55-Tps1A; (ii) pCY02M-Tps1G-Tps1H-Tps1A, pCY01M-Tps1B-Tps1C-Tps1D-Tps1E and pCY03M-Tps1J-Tps1K-Tps1L; (iii) pCY02M-Tps1G-Tps1H-Tps1A and pCY01M-Tps1B-Tps1C-Tps1D-Tps1E; (iv) pCY02M-Tps1H-Tps1A; and (v) pCY02M-Tps1H-Tps1A and pXW06-Tps1D. The transformants were inoculated into the corresponding 2 ml yeast synthetic drop-out medium and incubated with shaking at 250 rpm and 28°C for two days. A 20 μl aliquot of the seed culture was inoculated into 2 ml YPD medium (10 g l^−1^ yeast extract, 20 g l^−1^ peptone and 10 g l^−1^ glucose) and cultured at 28°C and 250 rpm for 5 days. The cultures were harvested and separated into cells and medium parts by centrifugation. For LC-MS analysis, the cells were extracted with acetone and concentrated *in vacuo*. The cell extracts were then dissolved in MeOH and analysed by LC-MS as described in Chemical analysis. For GC-MS analysis, the yeast culture was directly subjected to GC-MS analysis using the SPME method.

### (d) Biotransformation of the ^13^C-labelled substrate into Tps1A-expressing yeast

The *S. cerevisiae* strain harbouring pXW55-Tps1A was cultivated on 3 l of YPD at 28°C for 24 h with constant shaking at 240 rpm at 28°C. The culture media were transferred to an autoclaved conical centrifuge tube and centrifuged at 3750 rpm for 15 min. The supernatant was discarded, and the cell pellets were resuspended with YPD to obtain a final volume of 90 ml. The resuspended cell culture was divided into 30 ml aliquots in three 125 ml flasks. Each flask contained 246.0 mg of 1-^13^C-sodium acetate to reach a final concentration of 100 mM, and the mixture was incubated for 3 days with constant shaking at 240 rpm at 28°C. The cultures were centrifuged at 3750 rpm for 15 min. The cell pellets were extracted with acetone three times. The acetone extracts were concentrated *in vacuo,* and the residual water was then partitioned with hexanes three times. The hexane-soluble fraction was concentrated *in vacuo* to give the hexane extracts (58.5 mg) and then separated by a silica gel 60 column (230–400 mesh, 2 × 20 cm) eluted with 100% hexane (200 ml), 5% acetone/hexane (100 ml) and 20% acetone/hexane (200 ml) to give three fractions (fractions F1–F3). Fraction F2 was subjected to a semi-preparative RP-C18 high performance liquid chromatography (HPLC) column (Luna®, 5 μm phenyl-hexyl 100 Å, 250 × 10 mm) and eluted with 70% methanol/water (0–18 min), 70–95% methanol/water (18–30 min) and 95% methanol/water (30–60 min) with 1% formic acid at a flow rate of 1.0 ml min^−1^ to give Compound **^13^C-3** (1.1 mg, colourless amorphous solid, *R*_t_ = 21.0 min, PDA detector *λ*_m_ = 252 nm) (electronic supplementary material, scheme S5).

### (e) Overexpression and purification of His6-tagged Tps1D from *Saccharomyces cerevisiae*

A 2.5 ml aliquot of the *S. cerevisiae* RC01 strain harbouring pXW02-Tps1D was inoculated into 1 l of YPD medium (10 g l^−1^ yeast extract, 20 g l^−1^ peptone and 10 g l^−1^ glucose). The cell culture was then incubated at 28°C with shaking at 250 rpm for 3 days. The culture was harvested and separated into cells and medium parts by centrifugation. The cells were resuspended in 70 ml lysis buffer (50 mM NaH_2_PO_4_, 150 mM NaCl, 10 mM imidazole and pH 8.0) and lysed twice using a cell disruptor (Constant Systems Ltd.) at 23 kpsi. The homogeneous mixture was centrifuged at 14 000 rpm for 1 h at 4°C and filtered through a 0.45 μm filter to remove cellular debris. Ni-NTA agarose resin (Thermo Fisher Scientific) was added to the supernatant (1 ml) of the sample, and the solution was rotated at 4°C for 16 h. Soluble proteins were purified by gravity-flow column chromatography with increasing concentrations of imidazole in buffer A (20 mM–250 mM imidazole, 50 mM Tris-HCl, 500 mM NaCl and pH 7.9). Purified proteins were concentrated, and buffer was exchanged into buffer B (50 mM Tris-HCl, 100 mM NaCl and pH 8.0) using an Amicon Ultra15 Centrifugal Filter Unit and stored in 10% glycerol. The purified Tps1D protein was analysed by sodium dodecyl sulfate-polyacrylamide gel electrophoresis (SDS-PAGE), and the yield was calculated to be 12.5 mg L^−1^ using the Bradford assay with bovine serum albumin as a standard.

### (f) *In vitro* assay of Tps1H with compound **1**

The 50 μl reaction mixture contained 100 mg ml^−1^ microsomal fractions of Tps1H, 100 μM compound **1**, 2 mM NADPH, NADPH regeneration system (Corning Gentest™) (2.5 μl of solution A and 0.5 μl of solution B), 5 mM MgCl_2_ and 100 mM Tris-HCl buffer (pH 8.0). The reactions were incubated for 16 h at 30°C and were then extracted by ethyl acetate (100 μl × 3). The extracts were evaporated, dissolved in methanol and analysed by LC-MS.

### (g) *In vitro* assay of Tps1D with compound **5**

The 50 μl scale reaction mixture contained 10 μM Tps1D, 2 μM **5** and 100 μM *S*-adenosyl methionine (SAM). The reactions were incubated for 16 h at 30°C and were then extracted by ethyl acetate (100 μl × 3). The extracts were evaporated, dissolved in methanol and analysed by LC-MS.

## 3. Results

### (a) The *tps1* and *tps2* biosynthetic gene clusters encode UbiA-type cyclases from *Antrodia cinnamomea* s27

**Figure 2.**
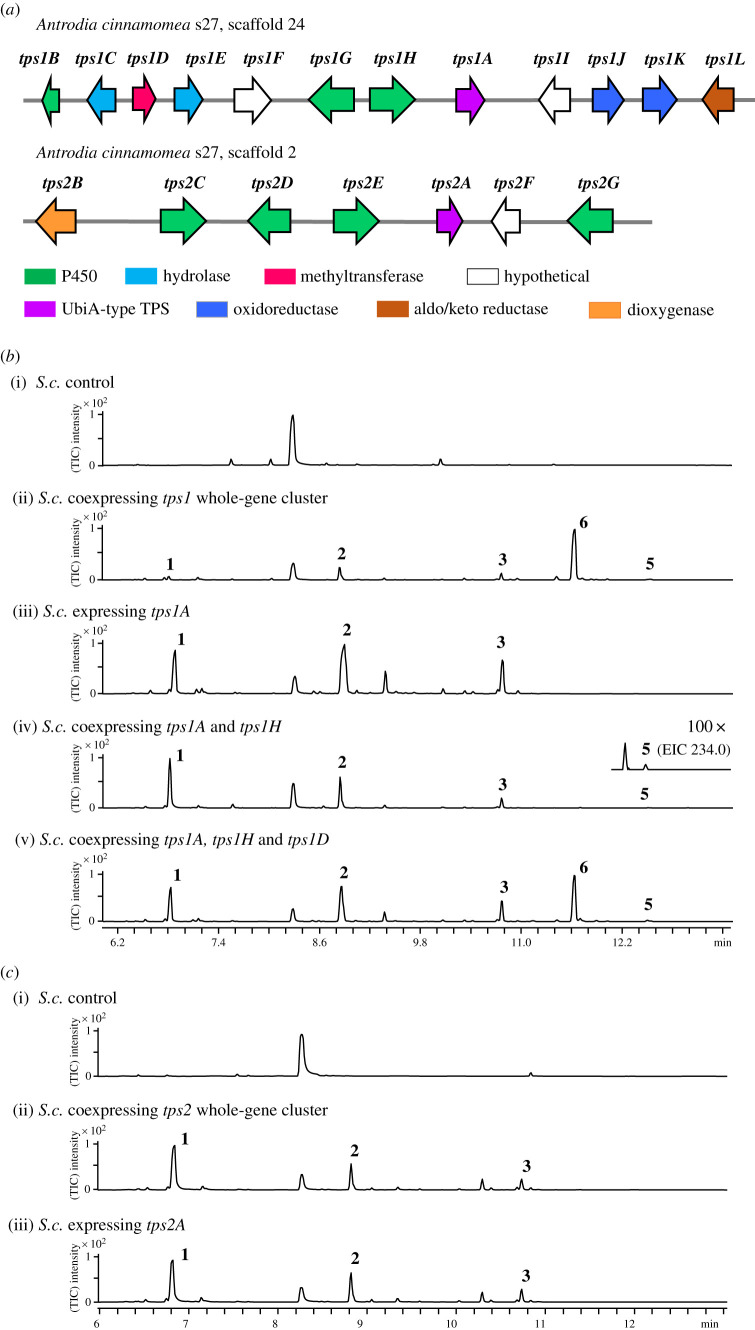
Heterologous expression of *tps1* and *tps2* BGCs in *S. cerevisiae* cultured for 5 days. (*a*) BGCs of *tps1* and *tps2* in *A. cinnamomea*; (*b*) SPME-GC-MS analysis of compounds produced through different combinations of *tps1* and (*c*) *tps2* genes. (Online version in colour.)

The UbiA superfamily of intramembrane prenyltransferases are involved in the biosynthesis of lipophilic metabolites, such as ubiquinones [32] and menaquinones [33,34]. A UbiA-type cyclase, Fma-TC from *Aspergillus fumigatus* Af293, containing a UbiA prenyltransferase domain, was characterized to convert FPP to *β*-*trans*-bergamotene [35]. To search for the UbiA-type cyclase homologues in *A. cinammomea*, we used Fma-TC as a query and BLAST against the genome of *A. cinnamomea* s27 from the NCBI database [28]. Two protein homologues, Tps1A and Tps2A, were located, and they shared 36%/47% and 28%/42% sequence identity and similarity to Fma-TC, respectively (and 50%/68% similarity to each other). These two UbiA-type cyclases are encoded in two biosynthetic gene clusters (BGCs), *tps1* and *tps2*, together with other associated enzymes that could modify the potentially terpene product scaffold (figure 2*a*).

### (b) Heterologous expression of *tps1* and *tps2* biosynthetic gene clusters in *Saccharomyces cerevisiae*

**Scheme 1.**
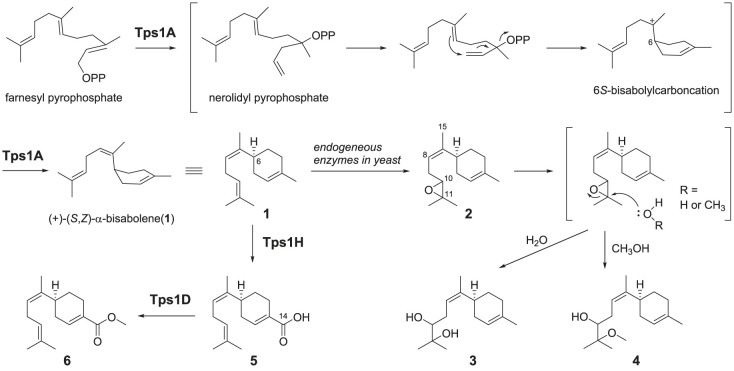
Proposed biosynthetic pathway to **6** through the combination of *tps1A*, *tps1H* and *tps1D* of the *tps1* BGC from *A. cinnamomea*.

To investigate the biosynthesis encoded by these two cryptic BGCs, we initially introduced all genes from *tps1* and *tps2* into *S. cerevisiae*. First, multiple gene expression vectors, pCY01M, pCY02M and pCY03M, were constructed. Each vector contained five auto-inducible P_ADH2_-like promoters (P_ADH2-*S. cerevisiae*_, P_ADH2-*S. bayanus*_, P_ADH2-*S. paradoxus*_, P_PCK1_ and P_MLS1_) that were acquired from the HEx yeast expression platform (electronic supplementary material) [36]. For the *tps1* BGC, the heterologous expression of the 10 intron-less genes (*tps1A*–*J*) in *S. cerevisiae* yielded new metabolites **1**–**3**, **5** and **6** compared to the host strain, as analysed by LC-MS and SPME-GC-MS (scheme 1) (figure 2*b*(i,ii); electronic supplementary material, figures S1 and S2). Large-scale cultivation of the transformed strain was performed, and **1**–**3**, **5** and **6** were purified together with an additional product **4** for structural elucidation. Compound **1** was characterized as (+)-(*S*,*Z)*-*α*-bisabolene (scheme 1) by comparing the optical rotation and NMR spectroscopic data (electronic supplementary material, figures S12–S17 and tables S6 and S7) to previous reports [5]. The ^13^C-NMR spectra of **2**–**4** showed characteristic signals of two oxygenated carbons at *δ* 64.0 and *δ* 58.0 of **2**, *δ* 79.5 and *δ* 72.7 of **3**, and *δ* 77.7 and *δ* 77.8 for **6** for C10 and C11, respectively. The nuclear overhauser effect spectroscopy data showed the key correlation of H8 to H15 (*δ* 5.20 to *δ* 1.65 for **2**, *δ* 5.30 to *δ* 1.62 for **3** and *δ* 5.30 to *δ* 1.62 for **4**), confirming the *cis*-configuration of the structure. Compounds **2**, **3** and **4** were characterized as (–)-(*S*,*Z)*-10,11-epoxy-bisabolene, (–)-(*S*,*Z)*-10,11-dihydroxy-bisabolene and (–)-(*S*,*Z)*-10-hydroxy-11-methoxy-bisabolene, respectively (electronic supplementary material, figures S20–S37 and tables S8–S11). For **5** and **6**, the ^13^C-NMR spectra showed characteristic signals of one carboxylic carbon at *δ* 171.1 (C14) on **5** and one methyl ester group at *δ* 167.7 (C14) and *δ* 51.6 (O-CH_3_) on **6**, respectively. Based on the two-dimensional NMR data and optical rotation, the structures of **5** and **6** were characterized as (–)-14-bisabolenoic acid and (–)-14-bisabolenoic methyl ester, respectively (electronic supplementary material, figures S38–S49 and tables S12 and S13).

For the *tps2* BGC, when co-expressing seven genes (*tps2A*–*G*) in *S. cerevisiae*, the production of **1**–**3** was observed by both SPME-GC-MS and LC-MS (electronic supplementary material, figures S1B and S2B). The NMR spectroscopic data and optical rotation data of these three products purified from the *tps2* co-expressing yeast strain were all identical to those purified from the *tps1* co-expressing strain (electronic supplementary material, figures S18, S19 and S58).

### (c) Characterization of Tps1A and Tps2A as (+)-(*S*,*Z*)-*α*-bisabolene synthases

To understand the function of *tps1A* and *tps2A*, the intron-less complementary DNAs of *tps1A* and *tps2A* were transformed into *S. cerevisiae*. The production of **1**–**3** was observed in both yeast strains expressing *tps1A* and *tps2A*, as analysed by SPME-GC-MS (figure 2*b*(iii)). To investigate whether Tps1A and Tps2A could accept longer isoprene pyrophosphate substrates, we co-expressed geranylgeranyl synthase (GGPPS) [31] for the C_20_ GGPP precursor with *tps1A* and *tps2A*, respectively. No additional product was observed compared to those when expressing *tps1A* and *tps2A* only (electronic supplementary material, figure S3). In addition, we prepared microsomal fractions from the yeast strain that overexpressed *tps1A* and *tps2A* for *in vitro* experiments. However, **1** co-purified with the microsome fraction, as the signal of **1** was still observed by SPME-GC-MS without the presence of FPP and MgCl_2_ (electronic supplementary material, figure S4).

**Figure 3.**
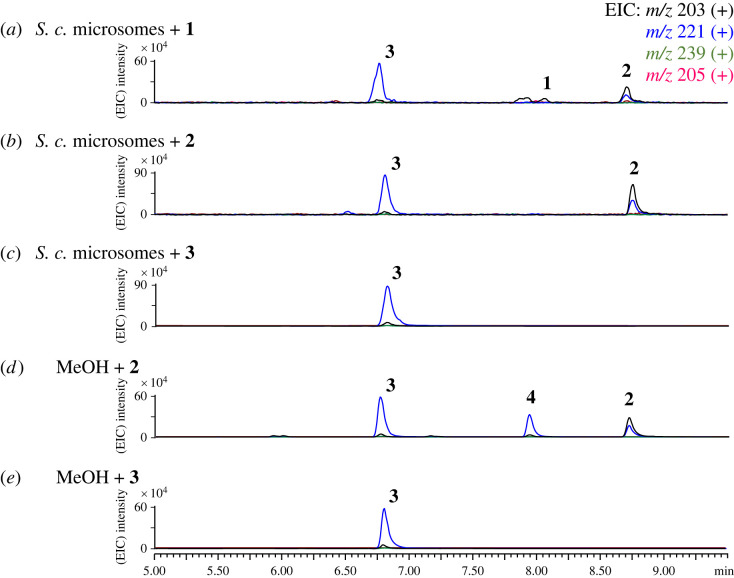
LC-MS analysis of the *in vitro* reaction of the microsome fractions of *S. cerevisiae* with (*a*) **1**, (*b*) **2** and (*c*) **3**. (*d*) Incubation of methanol with **2** and (*e*) **3** for 16 h. EIC, elective ionization. (Online version in colour.)

Next, the transformation of **1** into **2** and **3** involved an epoxidation reaction at C10 and C11 and a ring-opening reaction. To verify whether **2** and **3** could be the by-products of **1** generated in yeast, **1** was incubated with the microsome fractions of *S. cerevisiae*. The production of **2** and **3** was observed, indicating that the epoxidation of **1** was catalysed by endogenous enzymes from yeast (figure 3*a*). Incubation of **2** with the yeast microsome fractions led to the formation of **3**, while incubation of methanol with **2** led to the production of **3** and **4** (figure 3). These results indicated that **2** is a by-product generated from **1** in yeast and can be converted into **3** and **4** in the presence of water and methanol, respectively (scheme 1). The above results demonstrated that both Tps1A and Tps2A are (+)-(*S*,*Z*)-*α*-bisabolene synthases.

### (d) Verification of catalytic motifs on Tps1A and Tps2A

**Figure 4.**
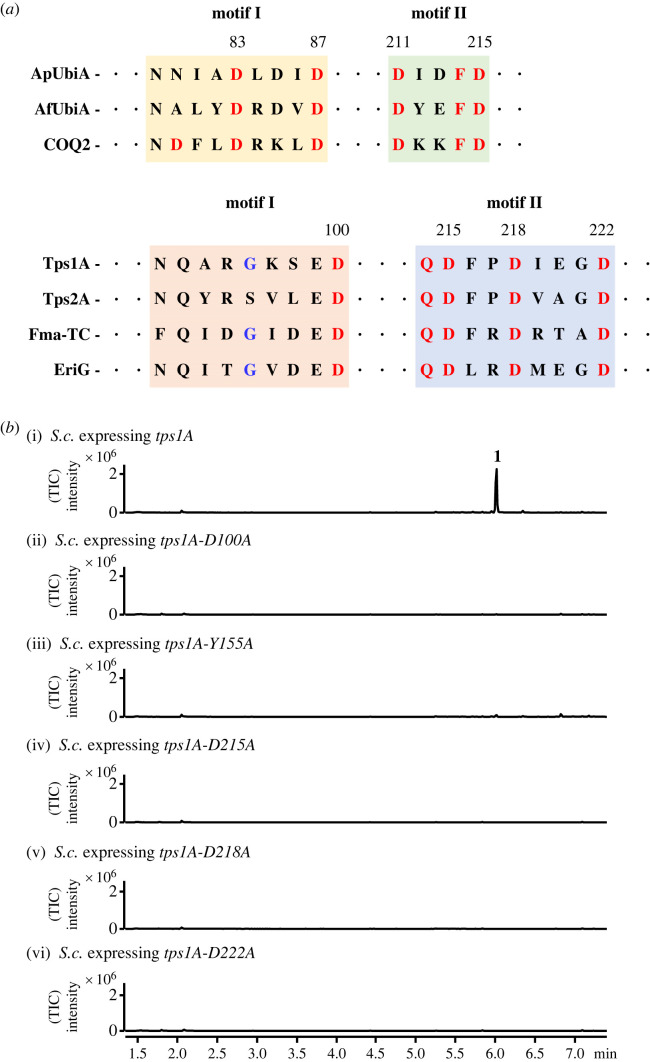
Verification of Tps1A active site motifs. (*a*) Alignment of amino acid sequences of the two conserved aspartate-rich motifs of the UbiA prenyltransferase family and the motifs of UbiA-like TPSs. (*b*) SPME-GC-MS analysis of yeast expressing the *tps1A* mutants D100A, D215A, D218A, D222A and Y155A. TIC, total ion content. (Online version in colour.)

The UbiA superfamily of intramembrane prenyltransferases, such as MenA from *E. coli* and COQ2 from *S. cerevisiae*, have two aspartate-rich motifs, NDXXDXXXD and DXXXD, as the first and second motifs, respectively. Although the first motif of the archaeal homologues ApUbiA [37] from *Aeropyrum pernix* and AfUbiA [38] from *Archaeoglobus fulgidus* differ from the consensus sequence of MenA and COQ2 (D_54_XXXD_58_ for ApUbiA and D_72_XXXD_76_ for AfUbiA, instead of NDXXDXXXD) (figure 4*a*; electronic supplementary material, figure S5), the crystal structures of ApUbiA and AfUbiA revealed the role of these two motifs in coordinating the Mg^2+^ ions that interact with the diphosphate of geranyl pyrophosphate or geranyl S-thiolodiphosphate. To detect the catalytic motifs of Tps1A and Tps2A, sequence alignment with their orthologous proteins revealed the presence of potential motif I as NXXX(G/A)XXXD on Tps1A and motif II as QDXXDXXXD, but not DXXXD like UbiA prenyltransferases, on both Tps1A and Tps2A (figure 4*a*). In addition, protein model prediction and alignment by the Phyre2 web portal [39] (based on template c4od5C, which belongs to 4-hydroxybenzoate octaprenyltransferase, a UbiA family protein from *Aeropyrum pernix* k1) suggested the coordination of three aspartate residues for Mg^2+^ binding on conserved motif II, QD^215^XXD^218^XXXD^222^ on Tps1A and QD^202^XXD^205^XXXD^209^ on Tps2A, respectively (figure 4*a*; electronic supplementary material figures S6 and S7). As Tps1A and Tps2A biosynthesized the same product **1** and shared a highly conserved QDXXDXXXD motif, we chose Tps1A for further mutagenesis studies. The Tps1A mutants D100A, D215A, D218A and D222A were constructed and expressed in *S. cerevisiae*. SPME-GC-MS analysis of the yeast strains expressing the above mutants showed that **1** was abolished (figure 4*b*). These results verified the crucial role of the aspartate residues on the conserved motif of both Tps1A and Tps2A in the cyclization of FPP to **1**. Furthermore, a highly conserved Y155 on Tps1A corresponds to Y139 on AfUbiA, which may involve the stabilization of allylic carbocation intermediates and stereospecific proton elimination by pyrophosphate. Indeed, the mutation of Y155 to A155 abolished **1** (figure 4*b*).

### (e) Generation of ^13^C-labelled products in *Saccharomyces cerevisiae* and the mechanism of Tps1A in biosynthesizing **1**

**Figure 5.**
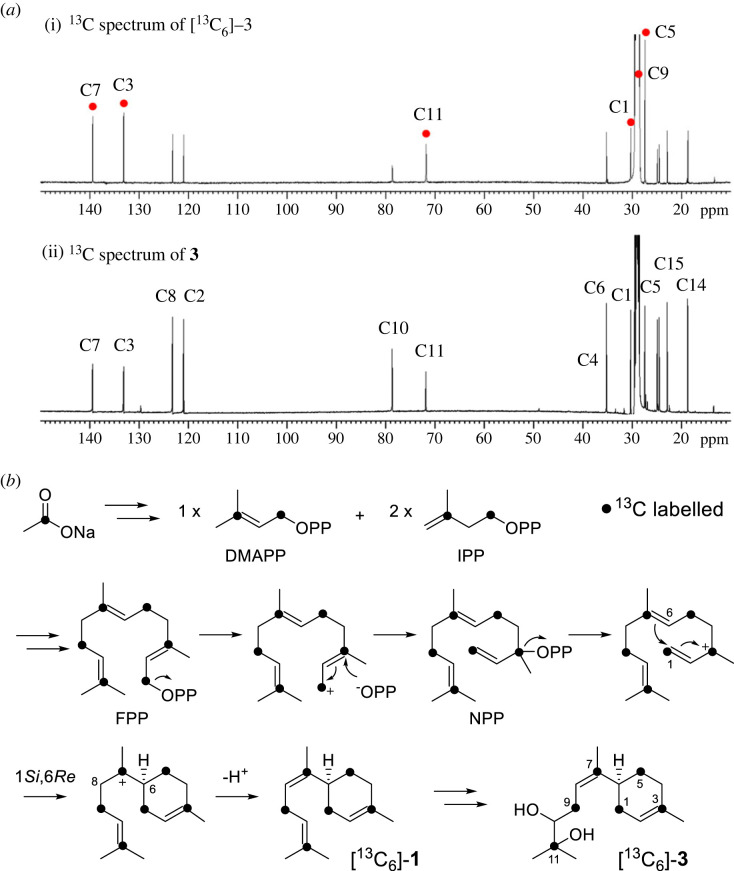
The labelling experiments with **[^13^C_6_]-3** using 1-^13^C-sodium acetate. (*a*) The comparison of the ^13^C-NMR spectra of (i) ^13^C-labelled and (ii) unlabelled **15**. (*b*) Proposed mechanism of the cyclization of FPP to **1** and generation of **3**. (Online version in colour.)

To examine the cyclization mechanism of **1** generated from Tps1A, 1-^13^C-sodium acetate was supplemented to the yeast strain expressing *tps1A* (electronic supplementary material). The isotopically labelled carbon was incorporated into specific positions of FPP, which was then converted into **[^13^C_6_]-1** and then led to the formation of **[^13^C_6_]-2** and **[^13^C_6_]-3** (electronic supplementary material, figure S8), as detected by SPME-GC-MS. To verify the position of the labelled carbon in the products, **[^13^C_6_]-3** was further purified from the 3 l yeast culture fed with 1-^13^C-sodium acetate after concentration 100 times. The ^13^C-NMR spectrum of **[^13^C_6_]-3** showed that the C1, C3, C5, C7, C9 and C11 signals were increased compared to those of **3**, indicating the position of carbons that were isotope labelled (figure 5). The results demonstrated that the cyclization of FPP to **1** proceeds through C1–C6 bond formation (figure 5*b*). This could be enabled by the initial isomerization of FPP to give nerolidyl diphosphate (NPP) [40–42]. Following ionization of NPP, 6*S*-bisabolyl carbocation intermediate is generated by Tps1A, which therefore determines the stereochemistry of C6 on **1**. Deprotonation at C8 generates the *cis-*configuration of **1** (scheme 1). The results indicated that Tps1A catalyses ionization-initiated cyclization, similar to Class I TPSs, even though its protein sequence is distinct from that of canonical Class I TPSs.

### (f) Determination of the minimal set of genes to form compound **5**

To understand the function of genes that biosynthesize **4** and **5**, different combinations of genes on *tps1* were transformed into *S. cerevisiae*. As the family of P450 monooxygenases are usually capable of performing C-H oxidation on the terpene scaffold and coded next to a terpene cyclase gene, we coexpressed *tps1A*/*G*/*H* with the possible post-modifying enzymes encoded by *tps1B*/*C*/*D*/*E* and *tps1J*/*K*/*L*, respectively. LC-MS analysis of the *tps1A*/*G*/*H* with *tps1B*/*C*/*D*/*E* strain showed no difference compared to the strain expressing *tps1A* only, while the *tps1A*/*G*/*H* with *tps1B*/*C*/*D*/*E* or *D*/*E* strain both showed the production of **5** and **6** (electronic supplementary material, figures S1 and S2). Next, upon coexpression of *tps1A*/*D*/*G* and *tps1A*/*D*/*H* in yeast, only the *tps1A*/*D*/*H* strain yielded **5** and **6** (figure 2*b*). The above results indicated that the minimal set of genes in the biosynthesis of **5** is terpene cyclase (Tps1A), P450 monooxygenase (Tps1H) and methyltransferase (Tps1D).

### (g) Verification of the functions of Tps1H and Tps1D

**Figure 6.**
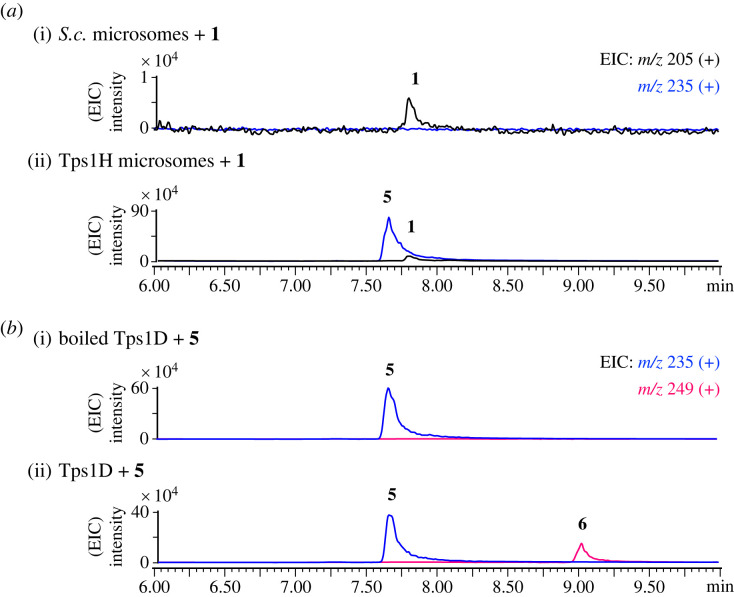
Verification of the function of Tps1H and Tps1D. LC-MS profiles of *in vitro* reactions of (*a*) Tps1H for the conversion of **1** to **5** and (*b*) Tps1D for the conversion of **5** to **6**.(Online version in colour.)

To verify the function of the P450 monooxygenase Tps1H, microsome extracts from the *S. cerevisiae* strain that overexpressed *tps1H* were prepared for *in vitro* characterization [31]. The incubation of microsomal Tps1H and **1** with nicotinamide adenine dinucleotide phosphate hydrogen (NADPH) yielded **5**, while no product was observed with the yeast microsomal extract control (figure 6*a*). The results demonstrated that Tps1H catalyses six-electron oxidation at C-14 to form the carboxylic group on **5**. To verify the function of methyltransferase Tps1D, recombinant Tps1D with a His6-tag from *S. cerevisiae* was purified into a soluble form (electronic supplementary material, figure S9). Incubating **5** with Tps1D and SAM led to the formation of **6** (figure 6*b*). The results confirmed the role of Tps1D in installing a methyl group on the 14-carboxylic group of **5** to form a methyl ester on **6**. Furthermore, the incubation of **1** with Tps1H and Tps1D together with SAM and NADPH led to the production of **6** (electronic supplementary material, figure S10). These results demonstrated the enzymatic synthesis of **5** from **1** in a one-pot reaction.

## 4. Discussion

**Figure 7.**
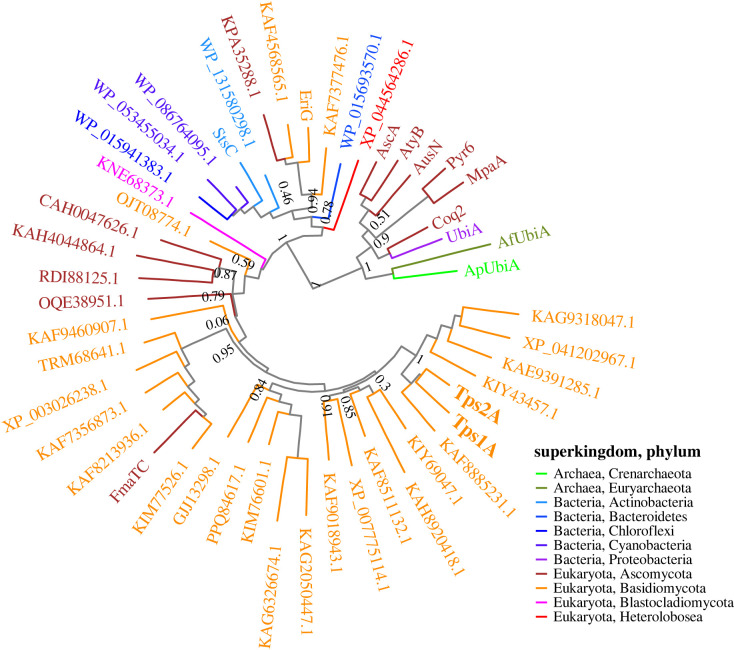
Phylogenetic analysis of Tps1A and Tps2A bisabolene synthases and UbiA-type TPSs, the UbiA superfamily of intramembrane prenyltransferases and integral membrane TPSs from different organisms.(Online version in colour.)

Bisabolene synthases from plants, such as *α*-(*E*)-bisabolene synthase (AgBIS), are soluble enzymes that contain conserved DDXXD and (N,D) DXX(S,T)XXXE metal-binding motifs, which are known to bind to divalent metal ions and trigger the cyclization of FPP through an ionization-dependent mechanism. Unlike canonical TPSs, the (+)-(*S*,*Z*)-*α*-bisabolene synthases Tps1A and Tps2A that we characterized here are membrane-bound UbiA-type TPSs. Sequence alignment of Tps1A and Tps2A to fungal *β*-bergamotene synthase (Fma-TC) and (–)-cyatha-3,12-diene synthase (EriG) showed that the NXXX(G/A)XXXD and QDXXDXXXD motifs are the conserved catalytic domains in these UbiA-type TPSs, and this was different from the NDXXDXXXD and DXXXD motifs, which are conserved in the UbiA prenyltransferase superfamily (figure 4*a*). Phylogenetic analysis of Tps1A and Tps2A with other UbiA prenyltransferases (e.g. AscA [43] which transfers FPP, and AtyB [44] which transfers DMAPP), integral membrane cyclases (e.g. Pyr6) and UbiA-type cyclases (e.g. Fma-TC [35], EriG [26] and StsC [45]) from different sources was performed. The results showed that they are distinct from the UbiA superfamily of intramembrane prenyltransferases and integral membrane cyclases (figure 7; electronic supplementary material, figure S11). In addition, Tps1A and Tps2A homologues formed a subclade adjacent to the Fma-TC-containing subclade and may belong to TPSs from Basidiomycota. Our study provided insights into the biosynthesis of bisabolenes in mushrooms that are generated by membrane-bound UbiA-type TPSs and demonstrated how nature has evolved different enzymatic tools to perform terpenoid biosynthesis.

Oxygenated bisabolane sesquiterpenes, todomatuic acid and its methylester juvabione from the genus *Abies* are known to suppress insect growth and reproduction (figure 1) [9,46,47]. Their structure mimics insect juvenile hormones and disrupts the activity of insects to moult into adults. Compounds **5** and **6**, generated from the heterologous reconstitution of *tps1* BGC in yeast, also contain the characteristic C14-carboxylic group and C14-methyl ester, which are found on todomatuic acid and juvabione, respectively. The *tps1* BGC contains several oxygenases such as P450s (Tps1B, Tps1G and Tps1H), and some reductases (Tps1J, Tps1K and Tps1L), which may participate to convert **1** into juvabione-like derivatives with Tps1H and Tps1D. The absence of further downstream products when coexpressed may be owing to the lack of other pathway enzymes or may be because some enzymes were not functional in *S. cerevisiae*. It is intriguing if the end pathway products of *tps1* BGC may participate in any mushroom–insect interaction. Alternative heterologous expression systems using other genus-closed strains deserve further investigation.

In conclusion, we identified two intramembrane UbiA-type sesquiterpene synthases, Tps1A and Tps2A, that convert FPP into (+)-(*S*,*Z*)-*α*-bisabolene (**1**). Four new bisabolene derivatives **2** and **4**–**6** were characterized from heterologous reconstitution in *S. cerevisiae*. In addition, two enzymatic machineries, a P450 monooxygenase (Tps1H) and a methyltransferase (Tps1D), were discovered and demonstrated to modify C14 of the bisabolene skeleton. Our study will provide opportunities to generate the chemical and structural diversity of bisabolene NPs using enzymatic approaches.

## Data Availability

Additional data are provided as the electronic supplementary material [48].
